# Classification tree analysis to enhance targeting for follow-up exam of colorectal cancer screening

**DOI:** 10.1186/1471-2407-13-470

**Published:** 2013-10-10

**Authors:** Yoshiki Ishikawa, Ying-Fang Zheng, Hiromu Nishiuchi, Takeo Suda, Tadahiko Hasumi, Hiroshi Saito

**Affiliations:** 1 Department of Public Health, Jichi Medical University, 3311-1 Yakushiji, Shimotuke-city, Tochigi 329-0498, Japan; 2 Cancer Scan, 1-18-1-6B, Dogenzaka, Shibuya-ku, Tokyo 150-0043, Japan; 3 Epidemiology Research Division Epidemiology Data Center, Japan Clinical Research Support Unit, Yushima D&A Bldg. 3 F 1-10-5 Yushima, Bunkyo-ku, Tokyo 113-0034, Japan; 4 DataScience Research Institute, 3-10-41-2 F, Minami-Aoyama, Minato-ku, Tokyo 107-0062, Japan; 5 Committee for Colorectal Cancer Screening of Omiya Medical Association, 2-107, Onari-cho, Kitaku, Saitama City, Saitama 331-8689, Japan; 6 Screening Assessment & Management Division, National Cancer Center, 5-1-1 Tsukiji, Chuo-ku, Tokyo 104-0045, Japan

**Keywords:** Colorectal neoplasms, Occult blood, Early detection of cancer, Patient compliance, Diagnostic examination, Classification tree analysis

## Abstract

**Background:**

Follow-up rate after a fecal occult blood test (FOBT) is low worldwide. In order to increase the follow-up rate, segmentation of the target population has been proposed as a promising strategy, because an intervention can then be tailored toward specific subgroups of the population rather than using one type of intervention for all groups. The aim of this study is to identify subgroups that share the same patterns of characteristics related to follow-up exams after FOBT.

**Methods:**

The study sample consisted of 143 patients aged 50–69 years who were requested to undergo follow-up exams after FOBT. A classification tree analysis was performed, using the follow-up rate as a dependent variable and sociodemographic variables, psychological variables, past FOBT and follow-up exam, family history of colorectal cancer (CRC), and history of bowel disease as predictive variables.

**Results:**

The follow-up rate in 143 participants was 74.1% (n = 106). A classification tree analysis identified four subgroups as follows; (1) subgroup with a high degree of fear of CRC, unemployed and with a history of bowel disease (n = 24, 100.0% follow-up rate), (2) subgroup with a high degree of fear of CRC, unemployed and with no history of bowel disease (n = 17, 82.4% follow-up rate), (3) subgroup with a high degree of fear of CRC and employed (n = 24, 66.7% follow-up rate), and (4) subgroup with a low degree of fear of CRC (n = 78, 66.7% follow-up rate).

**Conclusion:**

The identification of four subgroups with a diverse range of follow-up rates for CRC screening indicates the direction to take in future development of an effective tailored intervention strategy.

## Background

Colorectal cancer (CRC) is the second leading cause of cancer mortality in developed countries, with 727,400 new cancer cases and 320,100 deaths estimated to occur worldwide in 2008 [1]. As five-year CRC mortality rates vary according to the extent of tumor spread at the time of diagnosis, early detection is important.

Screening using the fecal occult blood test (FOBT) has been shown to reduce the incidence and mortality of CRC [2-7]. However, the potential benefit of screening for CRC has remained limited worldwide by failure to follow-up after FOBT. While compliance rates in a clinically controlled setting are over 80%, poor compliance rates ranging from around 30% to 70% have been reported in non-experimental settings [8-24]. Therefore, it is particularly important to develop effective intervention strategies to increase low post-FOBT follow-up rates.

Audience segmentation, which involves the identification of population subgroups that share particular characteristics, has been proposed as a promising strategy because interventions can be tailored toward particular subgroups [25-27]. Thus, segmenting the population could better guide the development of effective intervention strategies to increase follow-up compliance after screening tests. Specifically, segmentation can assist in the development of tailored interventions for high-risk subgroups with low follow-up rates, which have a high tendency to be undetected in existing mass screening programs.

Our study had two primary objectives: 1) to identify subgroups of individuals who share the same patterns of characteristics related to the follow-up exam after FOBT and 2) to examine the variance among identified subgroups in order to develop effective tailored interventions.

## Methods

### Setting

The study was conducted in the Omiya district of Saitama city in Saitama Prefecture adjunct to Tokyo, Japan. The population was 108,585 as of January 1st, 2010. During the period of the study, it was the local government’s policy to recommend an annual 2-day immunochemical FOBT screening for those aged 40 years and over. The FOBT is provided through a local medical association network of 170 clinics authorized by the local government. The local government informs eligible inhabitants about the screening once every year in April through pamphlets that are mailed to each household. Applicants then visit one of the 170 clinics to get the FOBT kit containing printed instructions for specimen collection and applicator sticks. Screening participants were required to conduct the specimen collection at home and to return the completed kits to the clinics. Participants were asked to visit the clinic again two weeks after undertaking the test to receive their diagnostic results. In the case of a positive result, participants were instructed by their physician to undertake additional tests.

### Procedure

Participants in this study were CRC screening participants recruited at the time they visited the clinic to get the FOBT kits. We handed letters requesting participation in the study to participants aged in their 50s and 60s. After obtaining oral consent to participate in the study, willing participants were asked to complete an anonymous questionnaire at home. The questionnaires were returned by the participants when they returned their FOBT kits to the clinic. The data collection period was from September 2009 to March 2010. The total number of CRC screening participants during the study period was 12,009.

### Participants

**Figure 1:**
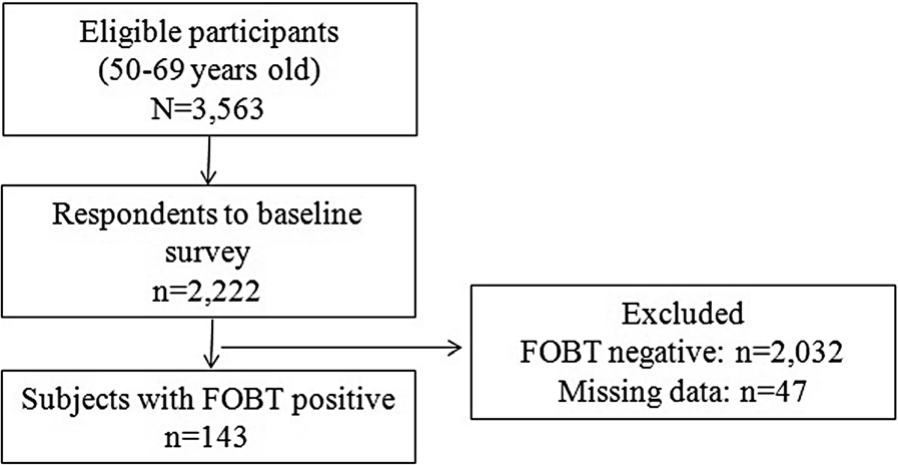
Participation flow.

Figure 1 shows the participation flow. Of the 3,536 participants who received the mail survey, 2,222 (response rate: 62.8%) replied. Following the baseline survey, 143 participants, who were asked to undergo follow-up examinations, were analyzed for the current study.

### Survey measures

Survey measures included a follow-up exam after FOBT as a dependent variable and sociodemographic variables, psychological variables, past FOBT and follow-up exam, family history of CRC, and history of bowel disease as predictive variables.

### Dependent variable

A follow-up exam after FOBT was employed as a dependent variable in this study. The number of follow-up exams was collected as a part of standard record-keeping of participating facilities. Each facility sent written notifications to the local government when a follow-up exam had been performed. This information was used to determine the number of follow-up exams.

### Predictive variables

Sociodemographic variables included age, sex, marital status, education, employment status, and subjective economic status.

The psychological variables used in this study were derived from the constructs of the Health Belief Model [28] and the Theory of Planned Behavior [29]. According to the Health Belief Model, a person’s behavior is determined by the following four beliefs: (a) perceived susceptibility, (b) perceived severity, (c) perceived barriers, and (d) perceived benefits. A previous systematic review suggested that the Health Belief Model is the most consistent model to predict CRC screening behavior [30]. Also, according to the Theory of Planned Behavior, a person’s behavior is driven by his/her intention to perform the behavior. For example, intention to undergo CRC screening has remained one of the strongest factors in past studies [31,32]. Accordingly, the psychological variables we measured in this study were the perceived susceptibility and severity of CRC, perceived benefits and barriers of follow-up exam after FOBT, and intention to undergo a follow-up exam. The measurements for these psychological variables were derived from a past study (see Zheng et al. [33] for detailed questionnaire).

Family history of CRC was assessed as a dichotomous (yes/no) variable with the statement “Have any of your first-degree blood relatives had CRC?”

Past CRC screening was assessed as a dichotomous (yes/no) variable with the statement “Have you ever undertaken an FOBT?” In addition, participants were asked whether they had ever received positive FOBT results and undergone follow-up exams.

### Statistical analysis

First, frequencies and percentages of measured variables are reported. Next, a classification tree analysis is performed in order to identify the best combination of the measured variables that predict compliance with follow-up exam after FOBT. Among multivariate statistical analyses, the classification tree analysis is suggested to be superior to cluster analysis or the logistic regression analysis in identifying distinctive homogeneous subgroups for further development of tailored intervention [34]. In the current analysis, we adopted chi-square values as a criterion for variable selection, and the groups were divided into two groups until the following criteria were met: (1) 10% or less of all participants after grouping or (2) no significant explanatory variables at p < 0.001. The outcome variable was follow-up exam after FOBT and the explanatory variables were socio-demographic variables, psychological variables, past FOBT and follow-up exam, family history of CRC, and history of bowel disease. Finally, in order to test differences between subgroups identified by classification tree analysis, ANOVA was performed on continuous variables and a Chi-square test on categorical variables. Measured variables were statistically tested and p < 0.002 was adopted as significance level by a Bonferroni correction. All analyses were performed using SAS 9.1.3 (SAS Institute, Cary, NC). Participants with missing data were excluded from the analysis.

### Ethical issues

This study was approved by the Institutional Review Board (IRB) of the National Cancer Center in Japan and adopted the principles of the Declaration of Helsinki.

## Results

### Baseline characteristics of respondents

Table 1 presents the characteristics of the study participants. The follow-up rate after FOBT was 74.1% (n = 106).

**Table 1:** Frequencies and percentages of measured variables

**Variable**			**n/Mean**	**%/SD**
Total			143	100.0
Follow-up exam			106	74.1
Socio-demographic characteristics				
	Age	50–59	36	25.2
		60–69	107	74.8
	sex	Male	63	44.1
	Marital status	Married	119	83.2
	Education	Less than high school	8	5.6
		High school	72	50.4
		Junior college/technical school	24	16.8
		College degree or higher	39	27.3
	Employment status	Employed	54	37.8
	Self-rated economic status	Poor/Somewhat affluent	26	18.2
		Average	98	68.5
		Affluent/Somewhat affluent	19	13.3
Family history of CRC		Yes	22	15.4
History of bowel disorder		Yes	71	49.7
Past FOBT screening		Yes	125	87.4
Past follow-up recommendation		Yes	34	23.8
Past follow-up exam		Yes	33	23.1
Psychographic characteristics				
	Intention	Yes	94	65.7
	Perceived benefits	6–30	25.6	(3.6)
	Perceived susceptibility	3–15	9.0	(2.7)
	Perceived severity	6–30	21.3	(4.8)
	Perceived barriers	13–65	38.7	(8.7)

### Classification tree analysis

**Figure 2:**
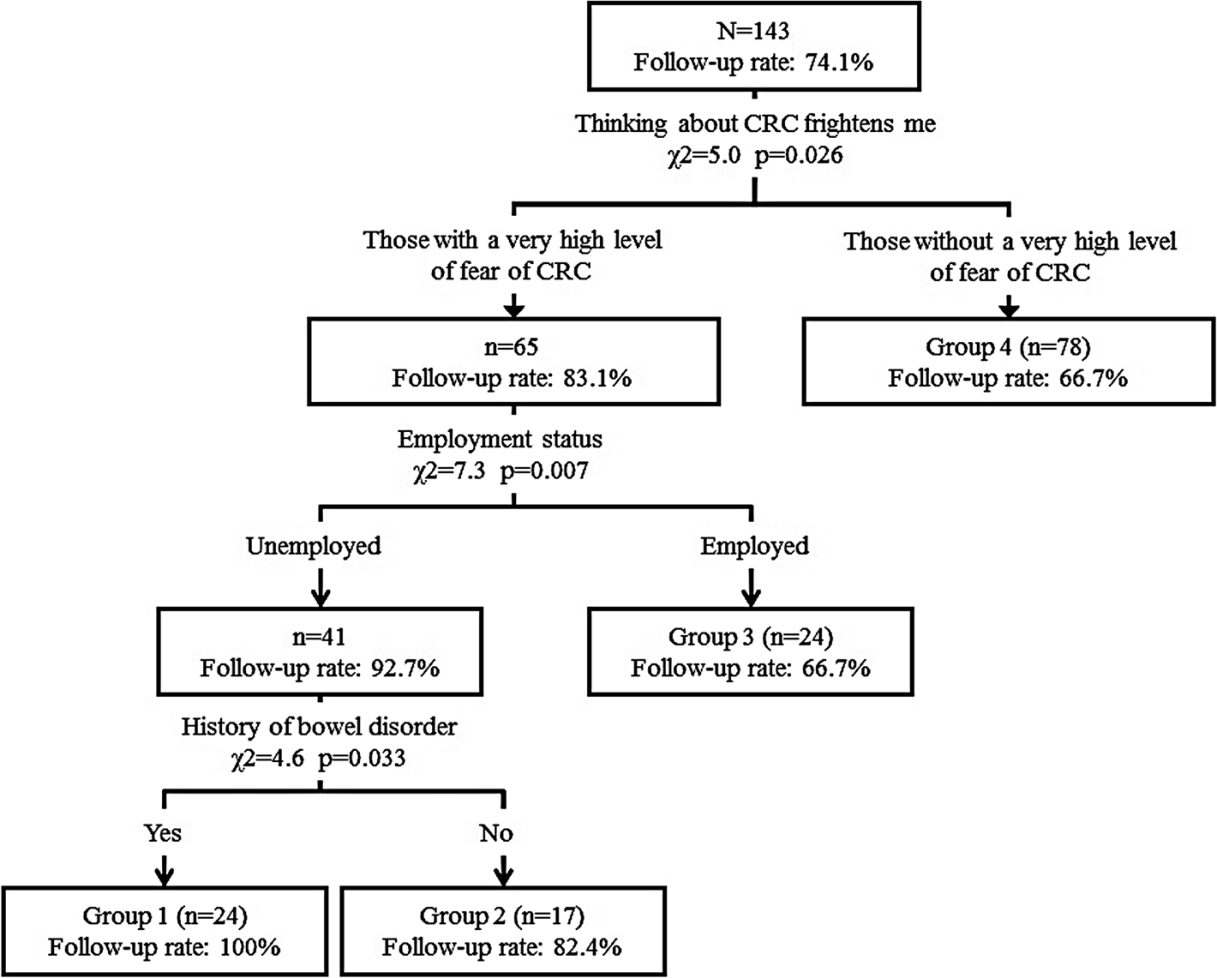
Classification tree analysis of follow-up exam after fecal occult blood test.

Figure 2 shows the result of the classification tree analysis. For all participants, the most appropriate explanatory variable that predicts compliance with follow-up exam after FOBT was fear of CRC. The was further classified into 2 groups: one with a high degree of fear of CRC (n = 65, 83.1% follow-up rate) and one with a low degree of fear of CRC (n = 78, 66.7% follow-up rate). The next most appropriate explanatory variable detected in the subgroup with a high degree of fear of CRC was employment status. This subgroup was further divided into two subgroups of unemployed (n = 41, 92.7% follow-up rate) and employed individuals (n = 24, 66.7% follow-up rate). On the other hand, for the subgroup with a lower degree of fear of CRC, no appropriate explanatory variable meeting the criteria was detected. Finally, the unemployed subgroup was divided into two subgroups of individuals with a history of bowel disease (n = 24, 100.0% follow-up rate) and those without a history of bowel disease (n = 17, 82.4% follow-up rate). At that point, the level of the criteria for the analysis completion was reached.

### Comparison of characteristics in each subgroup

Table 2 shows the characteristics of each subgroup identified by the classification tree analysis. There were statistically significant differences between subgroups in the following variables: sociodemographic variables such as education (p = 0.001) and employment status (p < 0.001); history of bowel disease (p < 0.001); and perceived severity (p < 0.001).

**Table 2:** Comparison among identified subgroups by classification tree analysis

			**Group 1**	**Group 2**	**Group 3**	**Group 4**	
**Variable**		**Range or category**	**% or mean (SD)**	**% or mean (SD)**	**% or mean (SD)**	**% or mean (SD)**	**p**
Follow-up exam		Yes	100	82.4	66.7	66.7	0.008
Socio-demographic characteristics							
	Age	50–59	20.8	23.5	33.3	24.4	0.767
		60–69	79.2	76.5	66.7	75.6	
	Gender	Male	29.2	35.3	41.7	51.3	0.222
	Marital status	Married	83.3	82.4	79.2	84.6	0.940
	Education	Less than high school	8.3	0.0	8.3	5.1	0.001
		High school	58.3	58.8	37.5	50.0	
		Junior college/technical school	25.0	29.4	37.5	5.1	
		College degree or higher	8.3	11.8	16.7	39.7	
	Employment status	Employed	0.0	0.0	100.0	38.5	<0.001
	Self-rated economic status	Poor/Somewhat affluent	16.7	29.4	8.3	19.2	0.309
		Average	66.7	70.6	83.3	64.1	
		Affluent/Somewhat affluent	16.7	0.0	8.3	16.7	
Family history of CRC		Yes	8.3	17.7	16.7	16.7	0.774
History of bowel disease		Yes	100.0	0.0	58.3	42.3	<0.001
Past FOBT screening		Yes	87.5	94.1	83.3	87.2	0.786
Past follow-up recommendation		Yes	20.8	29.4	29.2	21.8	0.809
Past follow-up exam		Yes	20.8	29.4	29.2	20.5	0.743
Psychographic characteristics							
	Intention	Yes	70.8	70.6	75.0	60.3	0.492
	Perceived benefits	6–30	25.5 (3.3)	25.4 (4.0)	26.3 (3.3)	25.5 (3.8)	0.805
	Perceived susceptibility	3–15	9.3 (2.7)	9.9 (3.0)	9.8 (3.2)	8.5 (2.4)	0.071
	Perceived severity	6–30	24.3 (2.3)	24.8 (3.7)	25.2 (3.6)	18.5 (4.1)	<0.001
	Perceived barriers	13–65	39.8 (10.3)	42.3 (8.2)	39.1 (8.2)	37.4 (8.3)	0.175

## Discussion

In order to achieve the goal of reducing colorectal cancer morbidity and mortality by mass screening, it is imperative that patients receive timely and appropriate follow-up exams for detected abnormalities. However, low follow-up rates after FOBT limits the potential benefit of mass CRC screening. Therefore, specifically from a public health perspective, targeting high-risk subgroups with low follow-up rates (i.e. people who are more likely to have CRC than the general public) is particularly important. This study is, to our knowledge, the first study to identify subgroups that share the same patterns of characteristics in terms of follow-up examinations after FOBT.

The most important finding of the present study is the identification of four subgroups with diverse follow-up rates (ranging from 66.7% to 100.0%) using classification tree analysis. This method has been shown to be a powerful medical decision-making tool [35]. Compared with cluster analysis or logistic regression analysis, the visual image of a hierarchical tree structure provides benefit to clinical practitioners, because the choice of a tailored message only depends on three questions: Fear of CRC, employment status, and past history of bowel disease.

A second implication is that fear of CRC, one of the psychological variables of perceived severity based on the Health Belief Model [28], has been demonstrated to have the closest association with follow-up examinations. Through selecting a combination of antecedent behavioral variables, the value of behavioral theories should be considered, as they could guide the development of effective intervention strategies [36]. The current limited research on examining the theory-based variables related to follow-up behavior after FOBT therefore calls for further focused and prospective research.

This study has several limitations. First, the sample size (n = 143) was small, and therefore the statistical power might be insufficient. Second, a selection bias should be considered in lieu of a relatively low response rate of 62.8%. Third, because the participants were recruited from a single urban community, generalization of the findings should be treated with caution. Fourth, not all confounders have been accounted for. Efforts to reduce chances for producing biases when segmenting the respondents, however, have been conducted as major confounders identified in the previous studies and were controlled statistically.

## Conclusions

We identified four subgroups of individuals who share the same patterns of characteristics related to their degree of compliance with the follow-up exam after FOBT. The unique characteristics of each identified subgroup suggest future development efforts to design an effective tailored intervention strategy.

## Competing interests

The authors declare that they have no competing interests.

## Authors’ contributions

YI was involved in design, interpretation of the data and drafting the manuscript. HS supervised the entire project and participated in the discussions on manuscript writing and finalization. YFZ assisted with the study design, literature review and questionnaire development. HN performed analysis of the data. TS and TH contributed to the development of the questionnaire and collecting data. All authors have read and approved the final manuscript.

## Pre-publication history

The pre-publication history for this paper can be accessed here:

http://www.biomedcentral.com/1471-2407/13/470/prepub

## References

[B1] Jemal A Bray F Center MM Ferlay J Ward E Forman D Global cancer statistics CA Cancer J Clin 2011 61 69 90 10.3322/caac.20107 21296855

[B2] Hardcastle JD Chamberlain JO Robinson MH Moss SM Amar SS Balfour TW James PD Mangham CM Randomised controlled trial of faecal-occult-blood screening for colorectal cancer Lancet 1996 348 1472 1477 10.1016/S0140-6736(96)03386-7 8942775

[B3] Jorgensen OD Kronborg O Fenger C A randomised study of screening for colorectal cancer using faecal occult blood testing: results after 13 years and seven biennial screening rounds Gut 2002 50 29 32 10.1136/gut.50.1.29 11772963 PMC1773083

[B4] Mandel JS Church TR Ederer F Bond JH Colorectal cancer mortality: effectiveness of biennial screening for fecal occult blood J Natl Cancer Inst 1999 91 434 437 10.1093/jnci/91.5.434 10070942

[B5] Hewitson P Glasziou P Irwig L Towler B Watson E Screening for colorectal cancer using the faecal occult blood test. Hemoccult Cochrane Database Syst Rev 2007 24 CD001216 10.1002/14651858.CD001216.pub2 17253456 10.1002/14651858.CD001216.pub2 PMC6769059

[B6] Saito H Soma Y Koeda J Wada T Kawaguchi H Sobue T Aisawa T Yoshida Y Reduction in risk of mortality from colorectal cancer by fecal occult blood screening with immunochemical hemagglutination test. A case–control study Int J Cancer 1995 61 465 469 10.1002/ijc.2910610406 7759151

[B7] Mandel JS Church TR Bond JH Ederer F Geisser MS Mongin SJ Snover DC Schuman LM The effect of fecal occult-blood screening on the incidence of colorectal cancer N Engl J Med 2000 343 1603 1607 10.1056/NEJM200011303432203 11096167

[B8] Kaye JA Shulman LN Screening program for colorectal cancer: participation and follow up HMO Pract 1991 5 168 170 10114296

[B9] Morris JB Stellato TA Guy BB Gordon NH Berger NA A critical analysis of the largest reported mass fecal occult blood screening program in the United States Am J Surg 1991 161 101 105 10.1016/0002-9610(91)90368-N 1987842

[B10] Myers RE Balshem AM Wolf TA Ross EA Millner L Screening for colorectal neoplasia: physicians’ adherence to complete diagnostic evaluation Am J Public Health 1993 83 1620 1622 10.2105/AJPH.83.11.1620 8238690 PMC1694870

[B11] Levin B Hess K Johnson C Screening for colorectal cancer. A comparison of 3 fecal occult blood tests Arch Intern Med 1997 157 970 976 10.1001/archinte.1997.00440300064005 9140267

[B12] Lurie JD Welch HG Diagnostic testing following fecal occult blood screening in the elderly J Natl Cancer Inst 1999 91 1641 1646 10.1093/jnci/91.19.1641 10511591

[B13] Sharma VK Vasudeva R Howden CW Colorectal cancer screening and surveillance practices by primary care physicians: results of a national survey Am J Gastroenterol 2000 95 1551 1556 10.1111/j.1572-0241.2000.02093.x 10894595

[B14] Shield HM Weiner MS Henry DR Lloyd JA Ransil BJ Lamphier DA Gallagher DW Antonioli DA Rosner BA Factors that influence the decision to do an adequate evaluation of a patient with a positive stool for occult blood Am J Gastroenterol 2001 96 196 203 10.1111/j.1572-0241.2001.03475.x 11197252

[B15] Myers RE Turner B Weinberg D Hyslop T Hauck WW Brigham T Rothermel T Grana J Schlackman N Impact of a physician oriented intervention on follow-up in colorectal cancer screening Prev Med 2002 137 132 141 10.1016/j.ypmed.2003.11.010 15020170

[B16] Ko CW Dominitz JA Nguyen TD Fecal occult blood testing in a general medical clinic: comparison between guaiac-based and immunochemical-based tests Am J Med 2003 115 111 114 12893396 10.1016/s0002-9343(03)00294-8

[B17] Turner B Myers RE Hyslop T Hauck WW Weinberg D Brigham T Grana J Rothermel T Schlackman N Physician and patient factors associated with ordering a colon evaluation after a positive fecal occult blood test J Gen Intern Med 2003 18 357 363 10.1046/j.1525-1497.2003.20525.x 12795734 PMC1494867

[B18] Baig N Myers RE Turner BJ Grana J Rothermel T Schlackman N Weinberg DS Physician reported reasons for limited follow-up of patients with a positive fecal occult blood test screening result Am J Gastroenterol 2003 98 2078 2081 10.1111/j.1572-0241.2003.07575.x 14499791

[B19] Nadel MR Shapiro JA Klabunde CN Seeff LC Uhler R Smith RA Ransohoff DF A national survey of primary care physicians’ methods for screening for fecal occult blood Ann Intern Med 2005 142 86 94 10.7326/0003-4819-142-2-200501180-00007 15657156

[B20] Etzioni DA Yano EM Rubenstein LV Lee ML Ko CY Brook RH Parkerton PH Asch SM Measuring the quality of colorectal cancer screening: the importance of follow-up Dis Colon Rectum 2006 49 1002 1010 10.1007/s10350-006-0533-2 16673056

[B21] Fisher DA Jeffreys A Coffman CJ Fasanella K Barriers to full colon evaluation for a positive fecal occult blood test Cancer Epidemiol Biomarkers Prev 2006 15 1232 1235 10.1158/1055-9965.EPI-05-0916 16775188

[B22] Garman KS Jeffreys A Coffman C Fisher DA Colorectal cancer screening, comorbidity, and follow-up in elderly patients Am J Med Sci 2006 332 159 163 10.1097/00000441-200610000-00001 17031239

[B23] Miglioretti DL Rutter CM Bradford SC Zauber AG Kessler LG Feuer EJ Grossman DC Improvement in the diagnostic evaluation of a positive fecal occult blood test in an integrated health care organization Med Care 2008 46 Suppl 1 91 96 10.1097/MLR.0b013e31817946c8 PMC4227983 18725839

[B24] Jimbo M Myers RE Meyer B Hyslop T Cocroft J Turner BJ Weinberg DS Reasons patients with a positive fecal occult blood test result do not undergo complete diagnostic evaluation Ann Fam Med 2009 7 11 16 10.1370/afm.906 19139444 PMC2625842

[B25] Slater MD Theory and method in health audience segmentation J Health Commun 1996 1 267 283 10.1080/108107396128059 10947364

[B26] Holt CL Shipp M Eloubeidi M Clay KS Smith-Janas MA Janas MJ Britt K Norena M Fouad MN Use of focus group data to develop recommendations for demographically segmented colorectal cancer educational strategies Health Educ Res 2009 24 876 889 10.1093/her/cyp024 19395624

[B27] Albada A Ausems MGEM Bensing JM Van Dulmen S Tailored information about cancer risk and screening: a systematic review Patient Educ Couns 2009 77 155 171 10.1016/j.pec.2009.03.005 19376676

[B28] Rosenstock IM Historical origins of the health belief model Health Educ Monogr 1974 1 8 10.1177/109019817800600406 299611

[B29] Ajzen I Kuhl J, Beckmann J From intentions to actions: a theory of planned behavior Action control: from cognition to behavior 1985 Berlin: Springer-Verlag 11 39

[B30] Kiviniemi MT Alyssa B Marie Z Marshal JR Individual-level factors in colorectal cancer screening: a review of the literature on the relation of individual level health behavior constructs and screening behavior Psychooncology 2011 20 1023 1033 10.1002/pon.1865 21954045 PMC3038178

[B31] Sutton SR Wardle J Taylor T McCaffery K Williamson S Edwards R Cuzick J Hart A Northover J Atkin W Predictors of attendance in the United Kingdom flexible sigmoidoscopy screening trail J Med Screen 2000 99 104 10.1136/jms.7.2.99 11002451

[B32] Watts BG Vernon SW Myers RE Tilley BC Intention to be screened over time for colorectal cancer in male automotive workers Cancer Epidemiol Biomark Prev 2003 12 339 349 12692109

[B33] Zheng YF Saito T Takahashi M Ishibashi T Kai I Factors associated with intentions to adhere to colorectal cancer screening follow-up exams BMC Public Health 2006 6 272 10.1186/1471-2458-6-272 17083746 PMC1664561

[B34] Kiernan M Kraemer HC Winkleby MA King AC Taylor CB Do logistic regression and signal detection identify different subgroups at risk? Implications for the design of tailored interventions Psychol Methods 2001 6 35 48 11285811 10.1037/1082-989x.6.1.35

[B35] Podgorelec V Kokol P Stiglic B Rozman I Decision trees: an overview and their use in medicine J med Syst 2002 26 445 463 10.1023/A:1016409317640 12182209

[B36] Glanz K Bishop D The role of behavioral science theory in development and implementation of public health interventions Annu Rev Public Health 2010 31 399 418 10.1146/annurev.publhealth.012809.103604 20070207

